# Developing an integrated genomic selection approach beyond biomass for varietal protection and nutritive traits in perennial ryegrass (*Lolium perenne* L.)

**DOI:** 10.1007/s00122-023-04263-8

**Published:** 2023-03-10

**Authors:** M. M. Malmberg, C. Smith, P. Thakur, M. C. Drayton, J. Wilson, M. Shinozuka, W. Clayton, C. Inch, G. C. Spangenberg, K. F. Smith, N. O. I. Cogan, L. W. Pembleton

**Affiliations:** 1 AgriBio, Centre for AgriBioscience, Agriculture Victoria Research, Bundoora, VIC 3083 Australia; 2 Hamilton Centre, Agriculture Victoria Research, Hamilton, VIC 3300 Australia; 3 School of Applied Systems Biology, La Trobe University, Bundoora, VIC 3086 Australia; 4 Barenbrug New Zealand, 2547 Old West Coast Road, Christchurch, 7671 New Zealand; 5 Faculty of Veterinary and Agricultural Sciences, The University of Melbourne, Melbourne, VIC 3010 Australia

## Abstract

**Key message:**

Breeding target traits can be broadened to include nutritive value and plant breeder’s rights traits in perennial ryegrass by using in-field regression-based spectroscopy phenotyping and genomic selection.

**Abstract:**

Perennial ryegrass breeding has focused on biomass yield, but expansion into a broader set of traits is needed to benefit livestock industries whilst also providing support for intellectual property protection of cultivars. Numerous breeding objectives can be targeted simultaneously with the development of sensor-based phenomics and genomic selection (GS). Of particular interest are nutritive value (NV), which has been difficult and expensive to measure using traditional phenotyping methods, resulting in limited genetic improvement to date, and traits required to obtain varietal protection, known as plant breeder’s rights (PBR) traits. In order to assess phenotyping requirements for NV improvement and potential for genetic improvement, in-field reflectance-based spectroscopy was assessed and GS evaluated in a single population for three key NV traits, captured across four timepoints. Using three prediction approaches, the possibility of targeting PBR traits using GS was evaluated for five traits recorded across three years of a breeding program. Prediction accuracy was generally low to moderate for NV traits and moderate to high for PBR traits, with heritability highly correlated with GS accuracy. NV did not show significant or consistent correlation between timepoints highlighting the need to incorporate seasonal NV into selection indexes and the value of being able to regularly monitor NV across seasons. This study has demonstrated the ability to implement GS for both NV and PBR traits in perennial ryegrass, facilitating the expansion of ryegrass breeding targets to agronomically relevant traits while ensuring necessary varietal protection is achieved.

## Introduction

Perennial ryegrass (*Lolium perenne* L.) is an economically important pasture crop worldwide, particularly in northern Europe, New Zealand, and Australia. Yet genetic gain has been limited, with biomass yield achieving 0.25–0.6% improvement on average per year (Wilkins and Humphreys 2003; Woodfield 1999) compared to a global average yield gain of 1.6% in maize, 1.0% in rice, 0.9% in wheat, and 1.3% in soybean (Ray et al. 2013). Many crop species are self-compatible, enabling trait fixation and making genetic improvement simpler for a wide range of favourable alleles. In contrast, ryegrass is an obligate outbreeding species with a self-incompatibility system resulting in high heterogeneity, increasing the difficulty of improving agronomically important traits. Ryegrass varieties are populations derived from a poly-cross of multiple parents and have greater variance within than between varieties (Bolaric et al. 2005; Guthridge et al. 2001; Wang et al. 2009). In addition, the average breeding program takes 10 years to complete, relying heavily on phenotypic selection (Lin et al. 2016; Wilkins and Humphreys 2003), often involving the evaluation of multiple generations including individuals cloned and transplanted into clonal rows, selection of multi-parent plant synthetic groups and subsequent evaluation through multiple generations as synthetic varieties. Further descriptions of a generic ryegrass breeding program can be found in Hayes et al. (2013).

A number of steps in a generic breeding program could be replaced with genomic selection (GS), primarily increasing genetic gain by shortening the breeding cycle (Lin et al. 2016). GS involves the use of a reference population which has been both genotyped and phenotyped to develop a prediction equation, which is then used to predict the phenotypes of samples which have only been genotyped (Meuwissen et al. 2001). This saves substantial time and resources typically required to perform phenotyping by allowing breeders to predict the performance of germplasm early on. Despite the inherent challenges presented by the ryegrass genome and population complexity, several studies have shown the potential of using GS for genetic improvement of ryegrass (Arojju et al. 2018; Faville et al. 2018, 2020) often using allele frequencies instead of bi-allelic genotypes in whole populations (Cericola et al. 2018; Fe et al. 2016; Guo et al. 2018; Keep et al. 2020; Pembleton et al. 2018). A study based on historical data in a breeding program has shown increased genetic gain would have been possible, had GS been applied through the breeding programs 15-year history, in comparison with the current phenotypic selection approach (Pembleton et al. 2018).

Several studies have shown GS in ryegrass is feasible and financially viable (Lin et al. 2017) but have mainly focused on yield-related traits or heading date (HD: Barrett et al. 2018; Byrne et al. 2017; Cericola et al. 2018; Faville et al. 2018, 2020; Fe et al. 2015; Guo et al. 2018; Pembleton et al. 2018), or crown rust resistance (Arojju et al. 2018; Cericola et al. 2018). All breeding programs primarily focus on yield and consider HD due to its importance in growth profiles and farm systems management as well as flowering time synchronicity. Nonetheless, ryegrass breeding programs need to incorporate new traits, with a greater focus on how and when these traits are measured, and their subsequent incorporation into breeding targets and selection indexes. It would be sensible to focus on largely un-targeted but valued traits such as nutritive value (NV), while simultaneously ensuring the protection of newly developed varieties through Plant Breeder’s Right (PBR) traits. As long as there is no consistent strongly negative correlation present, it is possible to simultaneously select for multiple traits using a weighted selection index.

Perennial ryegrass is a highly productive forage in terms of both yield and NV. However, a number of factors have prevented breeding for improved NV, primarily due to the expense and difficulty of measurement, but also due to the broad range of potential target traits, environmental effects, spatial and temporal variation. An examination across the history of Northern Ireland’s recommended perennial ryegrass varieties saw no consistent improvement in digestibility over time, but there was substantial variability between populations, primarily attributable to a set of specialist varieties bred for improved WSC, demonstrating that improvement is possible (McDonagh et al. 2016). This is promising for Australian cultivars, which also have not been selected for improved digestibility. Genetic gains for some NV traits may only be possible by selecting individuals rather than populations, such as cultivars or elite breeding lines, with crude protein (CP) and water-soluble carbohydrate (WSC) content in ryegrass cultivars showing substantial variability within, but not between, populations (Pembleton et al. 2016).

Understanding temporal fluctuation of NV is vital for pasture management decisions as NV is seasonal and at times of the year drops to the point that it creates a feed gap for dairy cattle (Machado et al. 2005; Redfearn et al. 2002) and is known to rapidly decline as the grass transitions to the reproductive phase just before summer dormancy (Waller and Sale 2001). NV has traditionally been measured through wet-chemistry or laboratory-based near-infrared (NIR) spectroscopy, both of which require destructive harvesting of the grass and are time-consuming and expensive (Smith et al. 1991). The development of cost-effective, in-field techniques in the form of reflectance-based in-field spectroscopy (Smith et al. 2019) allows the measurement of herbage quality over time and seasons. This will in turn provide correlations and guidance regarding the frequency of measurements needed to be able to breed for relevant NV traits, ultimately delivering varieties with higher NV. As mentioned, one method of incorporating NV improvements into other breeding targets is through GS, but to date only a small number of studies have assessed GS for NV, focusing either on families (Arojju et al. 2020; Fe et al. 2016; Grinberg et al. 2016; Skøt et al. 2018), primarily wild populations (Keep et al. 2020) or the effect of cutting time in a few cultivars (Wang et al. 2020).

While PBR traits in general have no agronomic value, they are a requirement plant breeders must take into consideration. Although a genetics-based classification of new cultivars would be optimal, currently to register a new cultivar ryegrass breeders must demonstrate distinctness, uniformity, and stability (DUS: UPOV 2006) based on several morphological traits including plant, leaf, and inflorescence characteristics (Wang et al. 2016a). These traits typically have simple genetic architecture and little agronomic importance. Although they can be modified relatively easily, this process of selection and screening for PBR traits increases the breeding cycle time and reduces the selection pressure for agronomically important target traits, ultimately limiting the rate of genetic gain. Simultaneous selection with other traits such as yield, without the requirement for specific and laborious screening nurseries using GS, would be highly beneficial. While most PBR traits have not been widely assessed (Keep et al. 2020), GS for HD has already been repeatedly demonstrated (Barrett et al. 2018; Byrne et al. 2017; Faville et al. 2020; Fe et al. 2015; Pembleton et al. 2018), suggesting that other PBR traits, which are also likely to be genetically simple with high heritability, will be well-suited to the method. Ultimately the benefit of GS is simultaneous selection for a range of traits, using a weighted selection index for traits targeted for improvement, such as biomass yield and NV, and restriction windows for PBR traits which need to be contained to within a nominated range when selecting parental groups to ensure DUS of resulting populations.

The aim of this study is to expand GS in ryegrass into a comprehensive suite of traits, to understand NV phenotyping requirements, and investigate the use of GS to modify NV and PBR traits. For NV traits, a single population of ryegrass, sampled across four seasonal timepoints, was tested for three important traits for overall NV: CP, WSC and in vitro dry matter digestibility (IVVDMD). For PBR traits, individual samples from clonal row nurseries with phenotypic data used in commercial breeding and collected across three years for five PBR traits described by UPOV was used: heading date (HD), leaf width (LW), height (H), leaf curvature (LC) and growth habit (GH).

## Materials and methods

### Phenotype data

#### NV

The NV trial used a population derived from a single variety consisting of 480 plants grown at Hamilton, Victoria, Australia and had phenotypes measured for three key NV traits: CP, WSC and IVVDMD. These plants were part of a larger trial that has been previously described in Gebremedhin et al. (2019), Smith et al. (2019) and Smith et al. (2020). Seasonal yield data for the same plants were obtained from Gebremedhin et al. (2019), and NV value data was obtained from Smith et al. (2020). Briefly, the plants were measured at the three-leaf growth stage, resulting in four timepoints in one year, May (MAY: Autumn), August (AUG: Early Spring), September (SEP: Early Spring) and November (NOV: Late Spring). At each timepoint, all plants were measured for canopy spectra and 64 plants had all herbage biomass removed higher than 5 cm from the ground surface and used for laboratory analysis as described in Smith et al. (2019) and to train the predictive model as described in Smith et al. (2020). Accuracies of predicted NV values using spectra can be found in Smith et al. (2020). The R function boxplot.stats (R Core Team 2018) was used to remove datapoints identified as outliers. The correlation matrix was generated in R using the ggpairs function of the R package GGally (Schloerke et al. 2018).

#### PBR

A number of PBR traits were phenotyped according to UPOV-described guidelines, with most characteristics visually classed from 1 to 9 except HD, which was recorded in days. For example, GH is scored from 1 to 9 for erect to prostrate growth, and LW is scored from very narrow to very broad using the 1 to 9 scale. Phenotypic data were collected for HD, LW and H in three years (2016, 2017, 2018), LC in one year (2016) and GH in two years (2017 and 2018) across a total of 5,026 clonal row samples. Briefly, 1,650 plants were sampled in 2016, 1,738 in 2017 and 1,638 in 2018. Plants were grown as clonal rows comprising five transplanted clonal copies (per genotype) within each row at Christchurch, New Zealand. Other than within row, the clonal row screening nursery was unreplicated.

### Genotype data

#### PBR and NV

For PBR samples RNASeq libraries were prepared from individual plants using the method described in Malmberg et al. (2018), while a modified in-house version of the same method was used for NV samples. For the modified in-house protocol, following mRNA extraction, polyA enrichment was performed again using Dynabeads™ (Life Technologies, Carlsbad, CA, USA) followed by random shearing using heat in the presence of Mg++. First-strand cDNA synthesis primed by random hexamers was performed using the Tetro Kit cDNA kit reverse transcriptase (Bioline, London, UK). Second strand was synthesised using DNA Polymerase I (New England Biolabs, MA, USA) and RNaseH (NEB) and ends were adenylated using Klenow (exo-) fragment polymerase (NEB). Inhouse PE-y PS adaptor was ligated to adenylated templates. Adaptor ligated templates were bead purified and then amplified using Phusion™ High-Fidelity DNA Polymerase (Thermo Fisher Scientific, Waltham, MA, USA) and barcoded PE Primers. Libraries were sequenced on a HiSeq 3000 platform (Illumina, San Diego, CA, USA), generating approximately 30 million reads (15 million paired) per clonal row sample and approximately 40 million reads (20 million paired) per NV sample. Sequencing reads were aligned to a perennial ryegrass transcriptome assembly (Shinozuka et al. 2017) using BWA-mem (Li and Durbin 2009). Genotypes at a pre-defined list of SNP loci (Malmberg et al. 2018) were called using bcftools mpileup v1.6 (Li et al. 2009). SNPs with alternative alleles other than those specified in the SNP list were removed. SNP loci with more than 25% missing data in samples with less than 50% overall missing data were removed before recalculating missing data and removing samples with greater than 90% missing data. Imputation was performed using the linkage disequilibrium k-nearest neighbour imputation (LD-kNNi) method (Money et al. 2015).

For NV, a total of 485 samples from a single population were genotyped. Initially 135,950 SNPs were called, with 108,232 SNPs remaining after removing SNPs with alternative alleles other than those specified in the SNP list. After filtering across SNPs and samples for missing data 481 samples remained with 62,095 SNPs and average missing data rate of 16.9% prior to imputation. Missing data was imputed with an estimated accuracy of 93.5%.

For PBR, a total of 5,026 clonal row samples were genotyped. Initially 197,137 SNPs were called, with 125,244 SNPs remaining after removing loci with alternative alleles other than those specified in the SNP list. After filtering across SNPs and samples for missing data 4,480 samples remained with 81,064 SNPs and average missing data rate of 26.2% prior to imputation. Missing data was imputed with an estimated accuracy of 93.7%.

### Genomic prediction

#### NV

Narrow-sense heritabilities were calculated in the R package BGLR (Pérez and de Los Campos 2014) using unscaled phenotypic data and performing heritability estimation using the sample variance of genomic values at each iteration of the sampler. Prior to heritability estimation, the genotype data were subset to contain only samples with available phenotype data, and SNP loci were further filtered for a minimum minor allele frequency (MAF) of 0.05. Estimated heritabilities were used as a prior for genomic prediction in all scenarios.

Genomic prediction was run using the R package BGLR (Pérez and de Los Campos 2014) using the BayesA (Meuwissen et al. 2001) model:

$${\varvec{y}}=u{1}_{n}+K{\varvec{v}}+{\varvec{e}}$$

where *y* is a vector of phenotype values for the trait of interest (i.e. heading date, crude protein, etc.), u is the population mean, 1_n_ is a vector of ones the same length, n, as the number of phenotypic values, K is a matrix of genotypes coded as the copy number of the alternative alleles (i.e. 0, 1, 2), *v* is a vector of random SNP effects estimated from the reference population, where each SNP effect is ${{\varvec{v}}}_{i} \sim N(0, {\sigma }_{{v}_{i}}^{2})$, and ${\varvec{e}} \sim N(0, {\sigma }_{e}^{2})$ is a vector of residual errors.

The BayesB model was also tested but found not to be significantly different (results not shown); therefore, in all analyses a BayesA model was fitted with a scaled-t density prior of marker effects. This was fitted with the default parameters used by the BLGR package. For computational convenience, BGLR samples marker effects from normal distributions where the variance of each SNP was sampled from an inverted Chi-squared distribution using default degrees of freedom and the scaling parameter defined by the BGLR package for trait heritability, with estimated heritabilities provided as described above. A total of 12,000 iterations with a burn-in of 2000 was used. Due to the relatively small scope of the NV study and using a single population, prediction accuracy was evaluated using a within-population k-fold approach, whereby each timepoint by phenotypic trait combination was evaluated by randomly masking 20% of samples at a time and averaged across the 5 iterations. Trace plots were manually investigated to ensure proper convergence of the algorithm. Genomic prediction accuracy was calculated as the correlation between GEBVs and observed phenotype.

#### PBR

Estimation of narrow-sense heritabilities and genomic prediction was performed with the same parameters as described for the NV data set. Estimated heritabilities were used as a prior for genomic prediction in all scenarios, with the exception of HD prediction using a reference population composed of synthetic varieties, where an expected heritability of 0.85 was used (Pembleton et al. 2018).

For PBR traits, it was possible to evaluate prediction accuracy using a number of scenarios including (1) within-population (population defined as the year) k-fold approach, whereby each year by phenotypic trait combination was evaluated by randomly masking 20% of samples, (2) forward prediction where the samples from previous years was used to predict into the next year, and (3) where a reference population composed of synthetic varieties from a breeding program (Pembleton et al. 2018) was used to predict the HD GEBVs for each year of advanced germplasm from the same program. Prior to each round of genomic prediction, the genotype data was subset to contain only samples with available phenotype data and SNP loci were further filtered for a MAF of 0.05.

In order to determine the effect of using GEBVs when grouping plants into 4-parent synthetic crosses, as compared to the currently used method based on observed PBR phenotypes, a simulation was run for various GEBV selection windows. The simulations were carried out in R by selecting the first parent plant randomly from the whole population. The remaining available samples were filtered to retain samples with a GEBV within the nominated selection window of the randomly selected first parent, and the second parent was randomly selected from among them. Remaining samples were once again filtered to only those within the nominated selection window of the first and second parents and a third parent randomly selected. This last step was repeated for the selection of the final parent. The range of phenotypes present within these simulated 4-parent synthetic groups was then calculated to determine how well GEBVs were able to restrict PBR phenotypes to within the expected range. This simulation was repeated 10,000 times, and the phenotype ranges were averaged.

## Results

### NV

#### Phenotype data and heritability

**Fig. 1:**
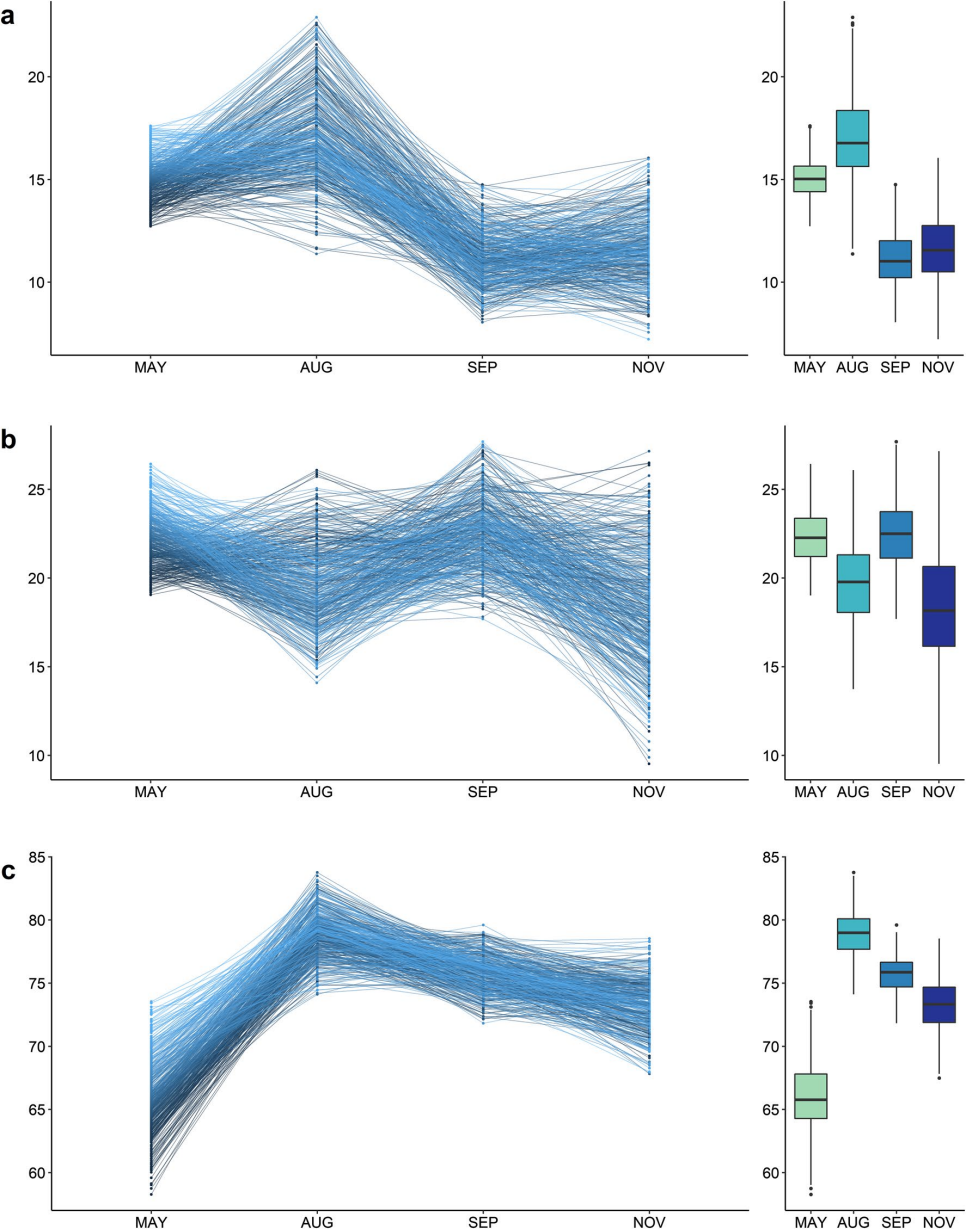
Phenotypic distribution of individuals across timepoints represented as line graphs on the left and as boxplots on the right for **a** CP, **b** WSC and **c** IVVDMD

The distribution of phenotypic data for NV traits is shown in Fig. 1 and was variable across timepoints, with nutritive composition of ryegrass changing with the seasons, typically decreasing into late spring. Examining individual samples across timepoints shows that seasonal fluctuation of NV traits is largely consistent across the whole population, but not between individuals, particularly for CP and WSC.

**Fig. 2:**
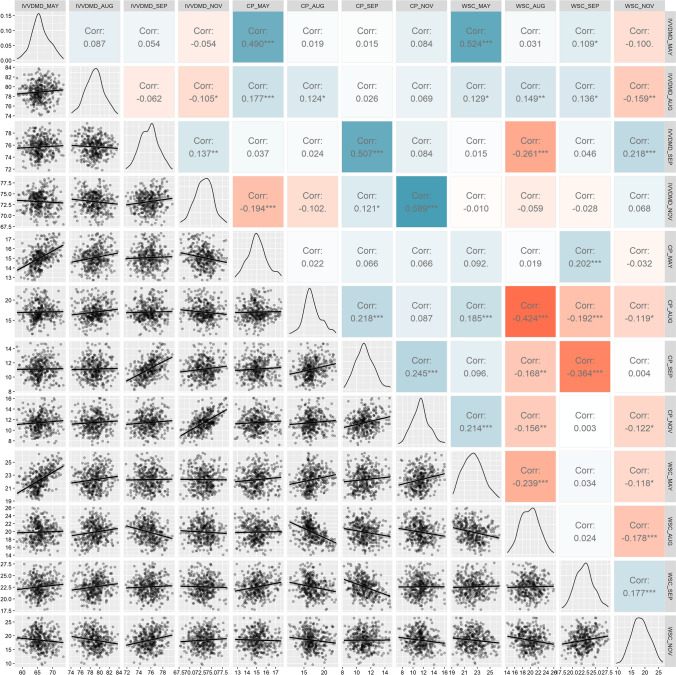
Correlation matrix showing phenotype correlations across time for IVVDMD, CP and WSC, with significance levels as follows: *p < 0.05; **p < 0.01; ***p < 0.001

Within traits, almost half (8/18) of the correlations between timepoints were significant (*p* < 0.05: Fig. 2) but varied between traits. Only SEP and NOV timepoints show significant but low correlation in all 3 traits. Consecutive timepoints were not always significantly correlated, suggesting that the nutritive profile of plants does not change consistently between individuals, as is also suggested in Fig. 1. Within timepoints, CP is significantly correlated with IVVDMD across all timepoints, while WSC is significantly correlated with IVVDMD for MAY (0.524) and AUG (0.149), but the correlation reduces across the four timepoints until it is no longer significantly correlated in SEP (0.046) or NOV (0.068). CP and WSC are not significantly correlated in MAY (0.092) but are strongly negatively correlated in AUG (− 0.424) and remain negatively correlated through SEP (− 0.364) and NOV (− 0.122), reducing over time. While early season WSC correlation with late season CP is generally low, there is significant correlation, and a similar trend is observed between early season CP and late season WSC. Some significant correlations are present between WSC and IVVDMD, but no clear pattern can be discerned. Across all timepoints there was no significant correlation found between either WSC, CP or IVVDMD and corresponding seasonal yield (data not shown). Narrow-sense heritability across traits and timepoints was low to moderate with the highest heritability for MAY CP at 0.43 and the lowest for NOV WSC at 0.20 (Table 1). There was a strong correlation between heritability and GS accuracy (0.85), which is expected as trait heritability determines the theoretical maximum of prediction accuracy ($\sqrt{{h}^{2}}$).

**Table 1:** Estimated narrow-sense heritabilities for NV traits

	CP	WSC	IVVDMD
MAY	0.429	0.294	0.372
AUG	0.284	0.385	0.231
SEP	0.325	0.352	0.266
NOV	0.214	0.201	0.270

#### Genomic prediction—within-population k-fold evaluation

Genomic prediction accuracies were low to moderate for NV, based on k-fold evaluation (Table 2). CP accuracies ranged from 0.184 to 0.474 across timepoints, while WSC and IVVDMD ranged from 0.041–0.345 and 0.153–0.394, respectively (Table 2). From a timepoint perspective, MAY had the highest average accuracies across all 3 traits (0.390), while NOV was the lowest (0.152).

**Table 2:** Mean GS accuracy evaluated using a k-fold within-population validation. Standard deviation in brackets

	CP	WSC	IVVDMD
MAY	0.474 (0.049)	0.303 (0.030)	0.394 (0.188)
AUG	0.258 (0.100)	0.312 (0.037)	0.274 (0.057)
SEP	0.226 (0.065)	0.345 (0.066)	0.153 (0.096)
NOV	0.184 (0.107)	0.041 (0.079)	0.232 (0.044)

### PBR

#### Phenotype data and heritability

**Fig. 3:**
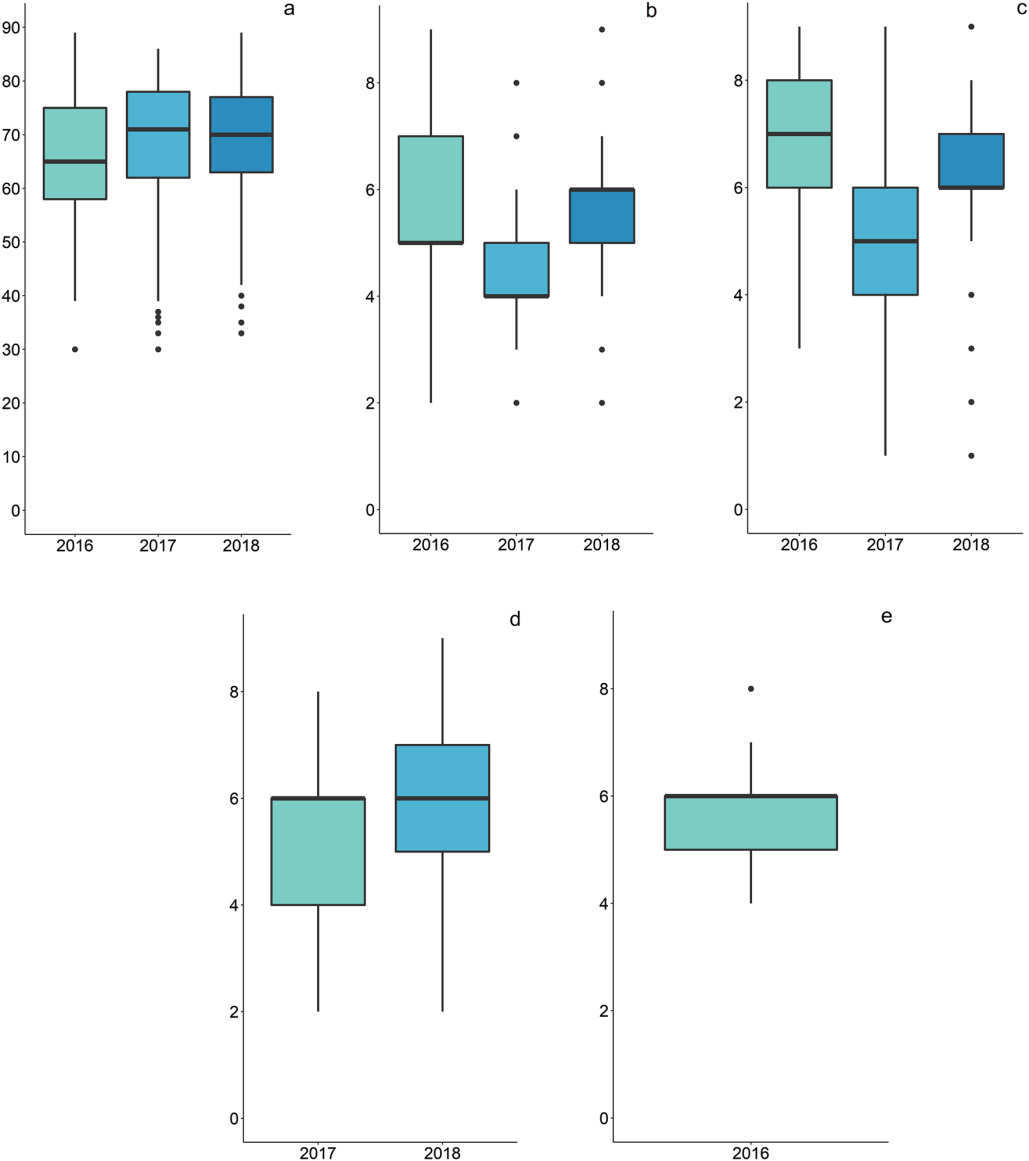
Box plots showing the phenotypic distribution of PBR traits across years for **a** heading date, **b** leaf width, **c** height, **d** growth habit and **e** leaf curvature

Phenotypic distribution was variable between years for most traits where multi-year data were available (Fig. 3). This was particularly the case for height, where phenotype distribution in 2017 had a significantly lower mean and larger range compared to 2016 and 2018 data. HD appears to have the most uniform phenotypic distribution between years, but it should be noted that this sample set does not contain common plants across the years, which would enable normalisation of the data across years.

Narrow-sense heritabilities for PBR traits were moderate to high (Table 3). Heritability was highest for HD (0.753–0.828) and lowest for LC (0.348). There was a strong correlation between heritability and GS accuracy (0.95).

**Table 3:** Estimated narrow-sense heritabilities for PBR traits

	HD	LW	H	LC	GH
2016	0.753	0.523	0.455	0.348	NA
2017	0.828	0.630	0.676	NA	0.605
2018	0.788	0.502	0.593	NA	0.583

#### Genomic prediction—within-population k-fold evaluation

Within-population evaluation of GS accuracy was moderate to high for PBR traits by year (Table 4). The highest accuracy was for HD 2017 (0.756), followed by HD 2018 (0.692). HD 2016 was lower but still relatively high (0.588). The lowest GS accuracy was for LC 2016 (0.250). H showed the most variability in GS accuracy between years (0.358–0.626).

**Table 4:** Mean GS accuracy for each of the three approaches used: k-fold within-population validation, forward prediction, and using a reference population consisting of synthetic varieties

	K-fold					Forward prediction				Synthetic RefPop
	HD	LW	H	LC	GH	HD	LW	H	GH	HD
2016	0.588 (0.027)	0.395 (0.070)	0.358 (0.049)	0.250 (0.037)	–	–	–	–	–	0.437
2017	0.756 (0.037)	0.598 (0.033)	0.626 (0.049)	–	0.560 (0.023)	0.578 (1.047)	0.300 (0.658)	0.052 (0.222)	–	0.620
2018	0.692 (0.036)	0.475 (0.010)	0.584 (0.033)	–	0.513 (0.022)	0.637 (0.855)	0.349 (0.590)	0.313 (0.539)	0.354 (0.547)	0.532

K-fold: Mean (sd)

Forward prediction: Mean (slope)

Synthetic RefPop: Mean

#### Genomic prediction—forward prediction

Using only data generated in previous years as the reference population to perform forward prediction (where multi-year data was available) delivered moderate-to-high GS accuracy in most traits (Table 4). An exception was when using 2016 data to predict H 2017, which had a GS accuracy of 0.052. All traits, except HD 2017 (1.047), showed inflated predicted values, again most notably in H 2017 (0.222) which had a low prediction accuracy. As expected, accuracies are reduced compared to a within-population evaluation.

#### Genomic prediction—synthetic varieties reference population for heading date

A set of 697 synthetic varieties from a breeding program with genotypes in the form of allele frequencies were used as the reference population to predict HD across all three years in individual clonal row samples. Similar to forward prediction, GS accuracy was reduced compared to within-population k-fold evaluation but remained moderate to high (Table 4).

#### GEBV selection window

In order to evaluate the application of the data in a breeding program to generate varieties that fit into the DUS categories, a set of selection windows were evaluated. The GEBV selection windows were based on the observed phenotypes in each data set, ranging from 0–89 for HD, 2–9 for LW, 1–9 for H, 4–8 for LC and 2–9 for GH. Using a smaller selection window marginally reduced the resulting mean phenotype range of simulated 4-parent groups (Table 5), but not to the same magnitude as expected, i.e. reducing the selection window by 1 unit did not reduce the phenotype range by 1 unit.

**Table 5:** Mean range of phenotypes in 10,000 simulated parental groupings for various GEBV selection windows

	GEBV selection window					
	1	2	3	5	6	7
HD	–	–	–	16.7	17.0	17.3
LW	2.9	3.1	3.4	–	–	–
H	3.0	3.2	3.5	–	–	–
LC	2.3	2.4	2.3	–	–	–
GH	3.2	3.3	3.5	–	–	–

## Discussion

Rapid methodological advancements enable a more comprehensive and sophisticated approach to breeding in ryegrass and will be essential in delivering an ongoing sustainable industry through superior outcomes and products. Routine use of modern technologies that have been developed and established in other species will be essential. Breeding programs have the potential to increase genetic gain threefold or more by implementing GS over traditional approaches (Lin et al. 2016; Pembleton et al. 2018), and the development of in-field reflectance-based phenotyping (Smith et al. 2019) enables expansion into traits that have previously been resource-intensive to phenotype.

### Phenotyping NV

Ryegrass is already highly valued as a forage crop due to its favourable NV profile, but this can be improved further (Faville et al. 2010; McDonagh et al. 2016; Muylle et al. 2013; Pembleton et al. 2016) to make livestock industries more sustainable and productive. This study has shown that in-field spectroscopy is a suitable method for high-throughput phenotyping of key NV traits, but further improvements in accuracy through expansion of reference populations and refinement of regression algorithms should be pursued. Current industry rankings, such as the Forage Value Index, take into consideration seasonal biomass yield as well as the target environment (Chapman et al. 2017) while factoring in seasonality, flowering time, ploidy, and overall persistence. The inclusion of metabolisable energy (ME) as a new trait in New Zealand rankings is an additional driver for selection in NV traits. Given the lack of correlation between seasonal yield and NV traits found in this study, consistent with other studies (McDonagh et al. 2016), NV is well suited for GS approaches alongside yield through the use of selection indexes.

To date, several factors have prevented the widespread pursuit of NV improvement in ryegrass breeding including the prohibitive cost of phenotyping, lack of consensus over traits to target (Chapman et al. 2015; Stewart and Hayes 2011), potential environmental effects (Arojju et al. 2020; Wang et al. 2016b), and variation in nutritional profile over plant lifecycle, seasons and for some traits even time of day (Fe et al. 2016; Wang et al. 2020; Wilkins and Humphreys 2003). The results of this study highlight that, like biomass yield, a seasonal selection approach will be required, primarily targeting the late season flowering period, as ryegrass plants commit resources to flowering over plant growth and create a feed gap for dairy cattle (Machado et al. 2005; Redfearn et al. 2002; Waller and Sale 2001). Further study is required to improve understanding of how NV changes across time and to determine optimal NV composition at different seasonal timepoints, which can then be targeted through selective breeding and GS to deliver genetic gain.

The results of the current study suggest that, in this population, CP contributes more consistently to overall digestibility, particularly later in the season as plants enter the flowering stage and may be linked to flowering time. Consistent with Pembleton et al. (2016), correlations between CP and WSC were often negative, such that it may not be possible to breed for increased WSC content without reducing CP content. This may be acceptable as Australasian varieties of ryegrass typically have an excess of CP based on animal requirements (Trevaskis et al. 2004). A ratio between CP and WSC may be more informative than either trait separately. An even more relevant measure would be ME, as the ultimate goal is to provide sufficient energy to dairy animals, and the inclusion of seasonal ME in selection indexes changes the ranking of ryegrass cultivars (Ludemann et al. 2018). Further research into which traits to target and when, the interplay between ryegrass traits as well as animal requirements, needs to be conducted in order to fully exploit the potential to improve overall productivity of ryegrass.

### Genomic prediction accuracy of NV traits

GS accuracy of NV ranged from low to moderate across traits and timepoints and was comparable to the accuracy achieved by Arojju et al. (2020) but lower than some other studies (Grinberg et al. 2016; Keep et al. 2020; Skøt et al. 2018). Heritabilities of these populations are likely to be the primary factor of the difference in GS accuracy. The current study and the Arojju et al. (2020) study have similar narrow-sense heritability estimates (0.20–0.43) and GS accuracies, while the other studies reported large broad-sense heritabilities for the NV traits examined, ranging from 0.41 to 0.74 (Grinberg et al. 2016; Keep et al. 2020; Skøt et al. 2018). Similarly, assessment of populations with high relatedness may account for the higher accuracies observed by Grinberg et al. (2016) and Skøt et al. (2018), who made use of half-sib families to predict the breeding values of mother plants. Although the use of GWAS-informed markers would account for some of the GS accuracy observed by Keep et al. (2020), the use of a single timepoint and populations rather than individuals may have resulted in inflated accuracies for NV traits due to the lack of variation between populations, as found previously (Pembleton et al. 2016). Consistently, the current study showed that on a population level, fluctuations of NV broadly follow a seasonal pattern, but individual genotypes display variability. There is only one breeding program, as far as we are aware, that has achieved and sustained population improvement of a nutritive trait, improving DMD through increased WSC content (Wilkins and Lovatt 2011). This was achieved partly or completely through population improvement breeding, which involves selection of individuals and their progeny families, reinforcing the assessment that selection of NV traits in individuals is a beneficial strategy for ryegrass breeding. However, there is a possibility that improved results would be obtained by using sward-based phenotypes to select individual plants, such as is the case for biomass yield due to the poor correlation between spaced individual plants and sward performance (Hayward and Vivero 1984; Waldron et al. 2008), making NV traits particularly suitable for GS breeding. With the exception of this single breeding program, NV traits have not been targeted by ryegrass breeding, so free segregation across populations is expected. As such, a lack of correlation between NV traits and yield is expected, as was confirmed in this study. Furthermore, the current study used elite, industry relevant germplasm rather than largely ecotypic material, and so may have higher relevance to breeding.

NV traits are likely to be under the control of many QTL with small effects (Arojju et al. 2020; Shinozuka et al. 2012), which may explain the reduced GS accuracies observed compared to PBR traits. As mentioned, heritability will also affect GS, limiting the maximum selection accuracy which can be achieved, and indeed this study observed a strong positive correlation between narrow-sense heritability and GS accuracy, as has been found in previous studies (Arojju et al. 2018, 2020; Crossa et al. 2017; Pembleton et al. 2018). The combination of a smaller population (481 vs 4480) and lower heritability for NV traits in comparison with PBR traits are likely to explain the difference in predictability. As such, GS of NV is likely to improve through the establishment of a larger reference population.

### Genomic prediction accuracy of PBR traits

GS accuracy was moderate to high for PBR traits, even with a range of prediction approaches applied, and although within-population evaluation was the most accurate, forward prediction as well as the use of a large reference population composed of synthetic varieties was found to be suitable and is more representative of the accuracy expected through ongoing GS implementation. This may be partly attributable to genetic architecture, as these traits are expected to be under control of few QTL with relatively large effects (Fe et al. 2015; Yamada et al. 2004), and Bayesian (variable selection) models have been found to also perform well when few loci contribute to genetic variation (Daetwyler et al. 2013). Accuracies for HD were comparable with that of other studies (Byrne et al. 2017; Cericola et al. 2018; Fe et al. 2015; Keep et al. 2020; Pembleton et al. 2018). In terms of less commonly studied PBR traits, in a study of mostly wild populations with some cultivars included, Keep et al. (2020) found GS accuracies of 0.64 and 0.80 for GH and LW, respectively, which is comparable to the mean prediction accuracy for GH (0.537) but a higher accuracy for LW than found in this study (0.489), which may be attributable to the inclusion of GWAS markers by Keep et al. (2020). Variability in the accuracy of phenotyping leaf and plant architecture traits by visually scoring may have contributed to the overall lower GS accuracy for PBR traits other than HD, rather than genetic architecture or GxE interactions.

The forward prediction approach resulted in reduced GS accuracies but remained moderate to high, with the exception of using 2016 data to predict H in 2017. Examination of the phenotypic distribution across the three years shows that 2017 had a significantly lower mean and larger range compared to 2016 and 2018, due to differences in either genetic effects, environmental effects or management conditions, highlighting the potential impact of genotype by environment (GxE) interactions on GS performance. Methods to minimise variation in sampling conditions should be considered, such as normalising data by including replicate samples across all testing years and incorporating GxE effects into the prediction model; however, prediction of average performance of PBR traits rather than performance under extreme environments is likely to be of greater value to breeding programs for the establishment of DUS.

Using a reference population composed of synthetic varieties from a breeding program to predict HD resulted in reduced prediction accuracies compared to within-population evaluation but remained moderate to high and comparable to forward prediction within clonal rows. Notably, the synthetic varieties reference population and the clonal row samples used in this study are derived from the same breeding program, and there is likely some genetic relationship between the two populations. This is beneficial for the application of GS within breeding programs, as it takes advantage of genetic relationships to improve persistency and accuracy of GS while leveraging existing resources, rather than developing large and relevant reference populations anew (Pembleton et al. 2018) but does come at the cost of decreased accuracy across generations and populations (Habier et al. 2007; Zhong et al. 2009).

Assessment of the use of GEBVs to select 4-parent groups from individual clonal row samples showed that the resulting mean phenotype range of simulated 4-parent groups did not change significantly with GEBV selection window. This may be attributable to the inflated prediction values observed, as GEBVs are higher compared to phenotypes such that while it appears there is a large difference in phenotype between samples based on GEBVs, this difference is not as pronounced in reality. The degree of variation, which is acceptable within a 4-parent group, will depend on the trait, and a possible strategy to mitigate this is to expand the groupings to include 5 or 6 parent plants based on GEBV and then use visual inspection to remove any plants which are obviously different in appearance. Although the creation of DUS populations could be improved through modelling of expected population distribution, GEBVs can be used to select 4-parent groups and achieve similar results to currently used phenotypic selection.

## Conclusion

Sensor-based phenomics are suitable for high-throughput phenotyping of NV traits in a GS context where screening of large diverse reference populations is required. The low-to-moderate accuracies achieved in this study are comparable to other studies and is expected to be improved through the expansion of reference populations, both for sensor-based phenotyping and for GS. Further consideration into which traits should be measured is essential, although it is clear that a seasonal approach is required as well as selection of individuals rather than populations for genetic improvement. Given the broad applicability of GS models to both complex and simple genetic traits, they are well suited to selection of PBR traits as demonstrated in this study. Furthermore, this study has shown that GEBVs can replace true phenotypes for selection of 4-parent groups for the generation of synthetic populations. Ultimately, PBR traits should base this selection on modelling the expected trait distribution in resulting synthetic populations. Nonetheless, applying restriction windows on GEBVs will achieve comparable results to the current phenotypic selection strategy typically employed by breeding programs. Modern technologies and breeding methodologies need to be fully exploited to further ryegrass breeding. This needs to begin with investing in the development of reference populations and prediction equations of numerous relevant traits to position strategically for the future.

## Declarations

### Data availability

NV: The datasets generated during and/or analysed during the current study are available from the corresponding author on reasonable request. PBR: The data that support the findings of this study are available from the Royal Barenbrug Group but restrictions apply to the availability of these data, which were used under license for the current study, and so are not publicly available. Data are, however, available from the authors upon reasonable request and with permission of the Royal Barenbrug Group.
